# Targeted and Untargeted Urinary Metabolomic Analyses of Organophosphate Pesticides Exposure and Attention-Deficit/Hyperactivity Disorder in Children

**DOI:** 10.1007/s11306-026-02495-5

**Published:** 2026-07-28

**Authors:** Hsin-Yun Tseng, Chi-Jen Lo, Boopathi Subramani, Jia-Woei Hou, Ching-Jung Yu, Ting-Yu Fang, Betau Hwang, Mei-Ling Cheng, Mei-Lien Chen

**Affiliations:** 1 Institute of Environmental and Occupational Health Sciences, School of Medicine, National Yang Ming Chiao Tung University, No. 155, Sec. 2, Linong Street, Taipei, 112 Taiwan; 2 Metabolomics Core Laboratory, Healthy Aging Research Center, Chang Gung University, No. 259, Wenhua 1st Road, Guishan Dist., Taoyuan, 333 Taiwan; 3 Clinical Metabolomics Core Laboratory, Chang Gung Memorial Hospital, Taoyuan, Taiwan; 4 School of Medicine, Fu Jen Catholic University, Taipei, Taiwan; 5 Department of Pediatrics, Cathay General Hospital, Taipei, Taiwan; 6 Department of Child and Adolescent Psychiatry, Taipei City Hospital, Taipei, Taiwan; 7 Department of Biomedical Sciences, College of Medicine, Chang Gung University, Taoyuan, Taiwan; 8 Graduate Institute of Biomedical Sciences, College of Medicine, Chang Gung University, Taoyuan, Taiwan

**Keywords:** OP pesticides, Neurodevelopment, ADHD, Oxidative stress, Metabolic profiling

## Abstract

**Introduction:**

Exposure to organophosphate pesticides (OPs) has been associated with increased oxidative stress and a higher risk of attention-deficit/hyperactivity disorder (ADHD). However, metabolic insights underlying ADHD and the potential pathophysiological role of OPs exposure remain limited.

**Objective:**

This study characterized urinary metabolomic profiles associated with ADHD and examined their associations with OPs exposure and oxidative stress.

**Methods:**

Urinary metabolites from 67 children with ADHD and 98 controls were analyzed using nuclear magnetic resonance (NMR) spectroscopy and ultra-perforamnce liquid chromatography quadrupole time-of-flight mass spectrometry analysis (UPLC-QTOF-MS). Dimethyl phosphate (DMP) and 4-hydroxy-2-nonenal-mercapturic acid (HNE-MA) were used as biomarkers of OPs exposure and oxidative stress, respectively. Children were classified into high- and low-exposure/concentration groups based on DMP or HNE-MA levels.

**Results:**

Urinary metabolomic profiles differed significantly between children with ADHD and controls. Several metabolites also differed between children with high and low DMP or HNE-MA levels. Metabolites involved in the tricarboxylic acid cycle were significantly higher in ADHD children and positively correlated with both DMP and HNE-MA. Pathway analysis suggested alterations in energy-related and amino acid metabolic pathways. Stepwise logistic regression and receiver operating characteristic curve analysis identified an 11-compound biomarker panel with good discriminatory performance in the discovery (AUC: 0.8450) and validation (AUC: 0.8748) stages.

**Conclusion:**

This metabolomic analysis suggests that OPs exposure and oxidative stress may be associated with metabolic changes in ADHD, particularly in energy metabolism and amino acid pathways. The identified biomarker panel may help distinguish children with ADHD from controls. Larger studies with multiple exposure assessments are warranted.

**Supplementary Information:**

The online version contains supplementary material available at https://doi.org/10.1007/s11306-026-02495-5.

## Introduction

Attention-deficit/hyperactivity disorder (ADHD) is one of the most prevalent neurobehavioral disorders in childhood, affecting approximately 5.9%–14% of children worldwide (Ayano et al., 2023; Faraone et al., 2021). ADHD is a multifactorial disorder involving genetic, biochemical, psychological factors, and environmental exposures that are thought to contribute to its etiology (Faraone et al., 2005; Poddar et al., 2025). However, the pathophysiological mechanism through which environmental exposure may contribute to ADHD remains unknown. Organophosphate pesticides (OPs) are among the environmental chemicals that have been increasingly implicated in ADHD development in children. Urinary dialkylphosphate (DAP) metabolites are commonly used as biomarkers of OPs exposure. These metabolites include three dimethyl alkylphosphate (DMAP) molecules, including dimethylphosphate (DMP), dimethylthiophosphate (DMTP), and dimethyldithiophosphate (DMDTP), and three diethyl alkylphosphate (DEAP) molecules, including diethylphosphate (DEP), diethylthiophosphate (DETP), and diethyldithiophosphate (DEDTP).

Epidemiological studies have reported associations between urinary DAP levels and neurodevelopmental outcomes. In the CHAMACOS cohort, higher urinary DAP levels were associated with increased odds of ADHD-related symptoms in children (Marks et al., 2010). Among DAP metabolites, DMP is widely recognized as the predominant metabolite of OPs. In Taiwanese children, our previous case-control study found a dose-response relationship between DMP levels and ADHD risk, with children in higher DMP exposure categories showing an approximately 2–3 fold increased risk of ADHD (Chang et al. 2021; Yu et al. 2016b). These findings are consistent with recent evidence suggesting that early-life chemical exposures, such as OPs, may contribute to ADHD risk, although the mecahnistic evidence remains limited and heterogeneous (Dimitrov et al., 2024). However, these DAP metabolites are considered biomarkers of exposure rather than mechanistic biomarkers. Therefore, additional biological markers are required to determine whether OPs exposure is associated with downstream biochemical changes related to ADHD pathophysiology.

Oxidative stress is one of the likely mechanisms linking OPs exposure with neurodevelopmental disorders. Exposure to OPs may cause neurotoxicity through oxidative stress, mitochondrial disorder, neuroinflammation, and other pathways (Chen et al., 2024). Lipid peroxidation is an important process in oxidative damage, and urinary 4-hydroxynonenal-mercapturic acid (HNE-MA) has been used as a biomarker of lipid peroxidation. A study by Chang et al. (2018) showed that the association between OPs exposure and ADHD is significantly influenced by oxidative stress, particularly lipid peroxidation, as indicated by elevated HNE-MA levels. In another study, we found a dose-response-based positive correlation between exposure to endocrine-disrupting chemicals 

and ADHD, with a significant contribution from HNE-MA, followed by DMP (Chang et al., 2021; Waits et al., 2022). These studies measured oxidative stress individually or in relation to genetic susceptibility and did not characterize metabolomic changes that may connect OPs exposure, oxidative stress, and ADHD-related pathways.

Metabolomics offers a powerful approach for identifying biochemical changes that reflect interactions among environmental exposures, endogenous metabolism, and disease conditions (Paglia et al., 2014). Metabolomic profiling can shed light on the biological pathways underlying complex neurodevelopmental disorders, as metabolites are downstream products of genetic, environmental, dietary, and physiological influences. Recent metabolomics studies have begun to identify metabolic signatures associated with ADHD (Predescu et al., 2024). Tian et al. (2022) explored the urinary metabolome of 76 ADHD patients and 363 controls and identified a biomarker panel involving tyrosine, leucine, and fatty acid metabolism that distinguishes ADHD from controls with good discriminatory performance. Wang et al. (2021) identified plasma biomarkers, such as guanosine and phenyl-leucine, distinguishing ADHD cases from controls using high-performance chemical isotope labeling (CIL)-based LC-MS. A study in Taiwan reported serum-based metabolomic differences between drug-naïve children with ADHD and controls, involving the gut-brain axis, dopaminergic function, oxidative stress, and purine salvage pathway (Hung et al., 2025). A systematic review found that metabolomic findings in ADHD are mostly linked to amino acid metabolism, neurotransmitters, and stress pathways (Predescu et al., 2024). There is still a significant research gap despite these advancements.

Most ADHD-associated metabolomic studies have focused solely on disease-related biomarkers, without considering any environmental exposure biomarkers such as OP metabolites. Meanwhile, studies on OPs exposure have explored the associations with neurobehavioral development or oxidative stress markers, but have not investigated metabolic changes in children with ADHD. A recent Spanish birth-cohort study found that urinary OP levels in the first trimester were associated with alterations in vitamin B6 metabolism, and later exposures were linked to disturbances in amino acid, neurotransmitter, and detoxification (Lopez-Flores et al., 2025). However, evidence remains scarce regarding whether early childhood exposure is associated with urinary metabolic alterations in ADHD children. Therefore, it is still unknown if OPs exposure and oxidative stress are associated with distinct urinary metabolomic profiles that could help in the explanation of ADHD-related biological disturbances.

The present study was designed to address this gap by using targeted and untargeted urinary metabolomics with OPs exposure and oxidative stress biomarkers in children with and without ADHD. DMP and HNE-MA were used as representative biomarkers of OPs exposure and oxidative stress. We postulated that children with ADHD, especially those with higher urinary DMP and/or HNE-MA levels, would have altered metabolomic profiles compared to control children. Therefore, this study aimed to generate mechanistic insight into whether urinary biomarkers of OPs exposure and oxidative stress were associated with metabolomic changes in children with ADHD under the exposure conditions during the study recruitment period of 2012–2015, identify metabolic pathways associated with ADHD in relation to OPs exposure and oxidative stress, and evaluate potential metabolite panels that can distinguish ADHD children from controls.

## Materials and methods

### Participants and data collection

The participants in this study were recruited from hospitals in Taipei City between 2012 and 2015. This recruitment period corresponds to our previously established case-control cohort designed to investigate the associations among OPs exposure, oxidative stress, genetic susceptibility, and ADHD (Chang et al. 2018; Yu et al. 2016a, b). The study adhered to the latest version of the Declaration of Helsinki. The institutional review board of a Taipei City hospital approved this study (IRB No. CGH-P101099, dated October 30, 2013, and March 31, 2014). Written informed consent was obtained from the participants and their parents prior to data collection. Detailed recruitment procedures have been published previously (Yu et al. 2016a, b).

Children with neurological deficits or intellectual disabilities were excluded. A total of 88 children with ADHD and 134 children without ADHD were included in this study. Parents or guardians completed a structured questionnaire that collected sociodemographic characteristics, including age, sex, BMI, children’s sports habits, parental education level, exposure to environmental tobacco smoke, maternal smoking, and alcohol consumption during pregnancy. Information on family history of neurological and neurodevelopmental disorders was also collected, including Parkinson’s disease, Alzheimer’s disease, ADHD, intellectual disability, cerebral palsy, autism spectrum disorder, epilepsy, developmental delay, multiple sclerosis, and peripheral neuromuscular diseases among grandparents, parents, or siblings of the participants. Spot urine samples were collected from the children and stored at ‒20 °C until analysis to minimize sample degradation.

For this metabolomics study, HNE-MA and DMP were selected as representative biomarkers of oxidative stress and OPs exposure, respectively, based on our previous studies reflecting the exposure patterns during the 2012–2015 study period (Chang et al. 2018; Yu et al. 2016b). To investigate urinary metabolic profiles in children with ADHD and without ADHD across different levels of DMP and HNE-MA, we conducted comprehensive urinary metabolite analyses using nuclear magnetic resonance spectroscopy (NMR) and ultra-high-performance liquid chromatography quadrupole time-of-flight mass spectrometry (UPLC-QTOF-MS) in both positive and negative ionization modes. To evaluate the reproducibility of the metabolomic analyses, samples were analyzed in two independent batches. The first analytical batch was designated as the discovery cohort, and the second as the validation cohort. The designation of study participants to the discovery or validation cohorts was based on the analytical workflow rather than clinical or demographic characteristics.

### NMR analysis

Urine samples were pretreated as described in a previous study (Dona et al., 2014). Briefly, aliquots of 180 µL of urine samples were mixed with 20 µL of NMR urine buffer (Bruker Biospin GmbH, Rheinstetten, Germany) containing 1.5 M K_2_HPO_4_/KH_2_PO_4_, 0.1% trimethylsilylpropanoic acid, and 2 mM NaN_3_ in D_2_O. The samples were then centrifuged at 12,000 rpm at 4 °C for 5 min, and 180 µL of the supernatant was transferred to a 4-inch SampleJet NMR tube for analysis. The NMR-targeted analysis evaluated 150 compounds on a Bruker Avance III HD 600 MHz NMR spectrometer (Bruker Biospin GmbH, Rheinstetten, Germany) equipped with a 5-mm inverse triple resonance probe (1 H/13 C/15 N) featuring a z-axis gradient and automated tuning and matching. Spectra were acquired by the Nuclear Overhauser Effect Spectroscopy (NOESY) pulse program with a relaxation delay of 4 s and a mixing time of 10 ms at 300 K. A total of 32 transients were acquired with 64k data points using a spectral width of 20 ppm for all urine samples. Data were processed using TopSpin (version 3.6, Bruker Biospin GmbH, Rheinstetten, Germany) software. Spectra were integrated over the range 0.3–10 ppm using AMIX software (version 3.9.14; Bruker Biospin GmbH, Rheinstetten, Germany). The region containing the water signal (4.7–4.95 ppm) was excluded, and the remaining variables were binned with a bin width of 0.01 ppm.

### UPLC-QTOF-MS analysis

Creatinine concentration was measured by UV spectroscopy and used to standardize urine concentration, thereby reducing the influence of variable urine dilution across spot urine samples. The creatinine concentration of each urine sample was adjusted to 100 µg/mL by adding urine buffer containing 0.1% formic acid in water. After centrifugation at 12,000 rpm at 4 °C for 30 min, the diluted samples were filtered through a syringe filter to obtain a clear solution for analysis. Samples were analyzed using an Ultra Performance Liquid Chromatography system (Waters ACQUITY UPLC System, Milford, MA, USA) coupled with a quadrupole time-of-flight mass spectrometer (Waters Synapt G1 HDMS System) (UPLC-QTOF-MS). Chromatographic separation was performed using a HSS T3 column (1.8 μm, 2.1 × 100 mm) (Waters Corp., Milford, USA) at 40 °C in both positive and negative ionization modes. The mobile phase consisted of 0.1% formic acid in water (Phase A) and 0.1% formic acid in acetonitrile (Phase B). The gradient was run at a flow rate of 0.5 mL/min over a total run time of 8.4 min, with a full-scan acquisition range of 50 to 1200 m/z. We used MassLynx 4.1 (Waters, Milford, MA, USA) to quantify peaks. The critical parameters for quantification included a mass tolerance of 0.03 Da, an intensity threshold of 50 counts, a mass window of 0.03 Da, a retention-time window of 0.1 min, and a noise elimination level of 6.0.

### Statistical analysis

All statistical analyses, except for multivariate analysis, were performed using SAS 9.4 Foundation. Student’s t-test was used for continuous variables, and the chi-square test was used for categorical variables to compare demographic characteristics between ADHD and control children, as well as between children in the discovery and validation cohorts. Because urine samples were standardized with creatinine concentration before UPLC-QTOF-MS analysis, creatinine was not included again as a covariate in the regression models. The grouping strategy was based on urinary HNE-MA and DMP levels among children with ADHD and controls. Urinary DMP concentrations in the first quartile were classified as the low-exposure group, while concentrations in the fourth quartile were classified as the high-exposure group. HNE-MA levels were grouped into high- and low-concentration groups, yielding four grouping methods (Table S1).

Multivariate analysis was performed using SIMCA-P 13.0 software (Umetrics, Umea, Sweden). Orthogonal partial least squares discriminant analysis (OPLS-DA) was used to determine whether features obtained from the UPLC-QTOF-MS analysis could distinguish between high- and low-exposure groups within the ADHD and control groups. Data analysis was preceded by Pareto scaling. Model quality was evaluated based on the cumulative variation in the Y matrix (R^2^Y) and the cross-validated predictive ability (Q^2^).

The ADHD group consisted of 88 participants, of whom only 13 were female. In the clustering analysis, the sample sizes for each group were too small; therefore, female-specific OPLS-DA was not performed. Triplicate measurements from the UPLC-QTOF-MS analysis were treated as separate samples for multivariate analysis. OPLS-DA was not performed for NMR-targeted data due to the limited number of detected compounds. The Mann-Whitney U test was performed on NMR data to compare metabolite concentrations between the low-exposure control and high-exposure control groups, and between the low-exposure ADHD and high-exposure ADHD groups. Spearman’s correlation was used to evaluate the relationships between urinary HNE-MA and DMP concentrations and individual metabolites.

### Pathway analysis and significant metabolite prediction panel development

Compounds identified in the NMR-targeted analysis were selected as significant if they had a *p*-value < 0.05 and a correlation coefficient > 0.3 or < -0.3 in any of the four comparison groups. The Kyoto Encyclopedia of Genes and Genomes (KEGG) database was used to identify pathways associated with these significant metabolites, followed by pathway integration, and plot generation using MetaboAnalyst 5.0 (Pang et al., 2021). Stepwise logistic regression was conducted to select the most significantly different metabolites between the ADHD and control groups for the predicted panel, with *p*-values set < 0.3 as an exploratory variable selection criterion. The *p*-value threshold of < 0.3 was used as a variable selection criterion to include all the potentially informative metabolites during model development. Statistical significance of the predictors in the final model was interpreted using the *p*-value < 0.05. The area under the receiver operating characteristic curve (AUC) was then calculated to assess model performance.

## Results

This study included 222 children after excluding participants with insufficient urine or creatinine levels outside the 30–300 mg/dL range. Demographic characteristics are presented in Tables S2 and S3. Among the 165 children in the discovery cohort, 67 were diagnosed with ADHD, and 98 were controls (Table S3). The mean age was approximately eight years, with a mean BMI of around 17 kg/m^2^. This study population included approximately twice as many boys as girls. Physical exercise habits were less common among children with ADHD than among controls. Most maternal and paternal education levels were generally college or higher, especially significantly higher in control children. A higher proportion of mothers in the ADHD group were exposed to second-hand smoke or consumed alcohol during pregnancy. In addition, family history of nervous diseases was more frequent in the ADHD group. No other demographic variables showed significant differences between the two groups. After normalization of urine samples to a creatinine concentration of 100 µg/mL, the median urinary levels were 35.31 ng/mL for HNE-MA and 40.60 ng/mL for DMP, with detection rates exceeding 95% for both biomarkers. Urinary concentrations of DMP (*p* < 0.001) and HNE-MA (*p* = 0.046) were significantly higher among ADHD children than in controls (Chang et al. 2021; Fang 2021; Subramani et al. 2023; Yu et al. 2016b).

**Fig. 1:**
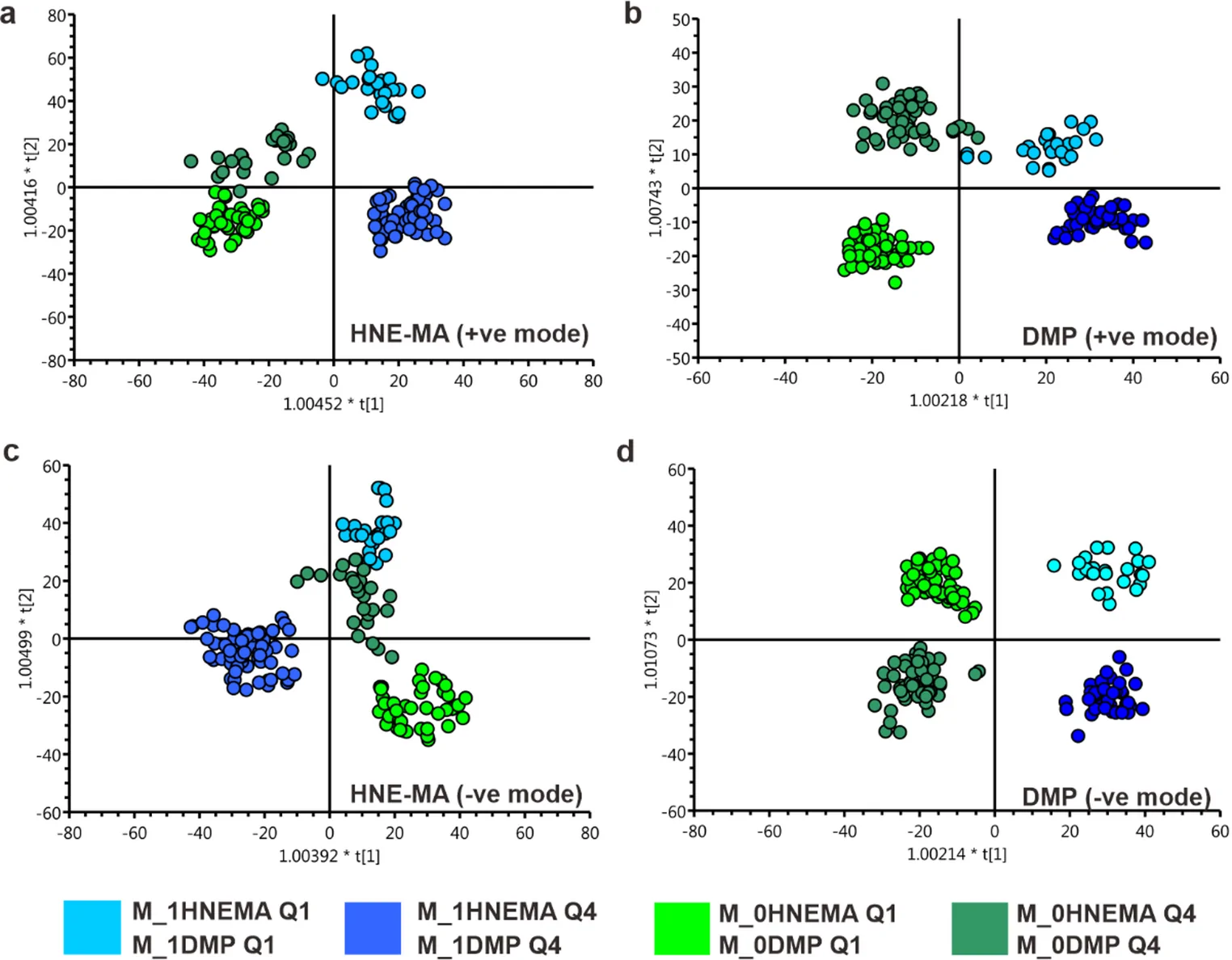
OPLS-DA score plots between case, control, low, and high exposure levels of DMP, and low and high concentrations of HNE-MA. Figures a-b and c-d represent the results of the positive charge and negative charge modes of LC-QTOF-MS, respectively. ‘M’ stands for male, ’0’ indicates control, and ‘1’ indicates case. ‘Q1’ indicates low exposure, which means exposure level < 25th percentile. ‘Q4’ indicates high exposure, which means exposure level > 75th percentile

The NMR-targeted analysis evaluated 150 compounds, of which 24 met the significance criteria. The fold changes in these compounds are summarized in Table 1. Most compounds showed higher concentrations in the ADHD group than in controls. The selected compounds exhibited positive correlations with urinary HNE-MA and/or DMP levels, with correlation coefficients greater than 0.3. In the UPLC-QTOF-MS analysis, 6,125 features were detected in positive ionization mode and 4791 in negative ionization mode. These features represent two-dimensional analytical data annotated by retention time and mass-to-charge ratio. Four grouping strategies were applied across positive and negative charge modes in UPLC-QTOF-MS. The Q2 and R2Y values in the OPLS-DA models exceeded 0.8, indicating a high degree of model fit (Table S4). OPLS-DA score plots were generated from these features to investigate the distinguishability of urinary metabolites among children with ADHD, controls, and those with low or high levels of HNE-MA and DMP. Because the clustering patterns were clear and consistent across models, four representative plots are presented in Fig. 1.

**Table 1:** Significant compounds in NMR-targeted metabolomic analysis

Compounds	FC^a^	FC^b^	FC^c^	Correlation coefficient^d^ (*R*)	
				HNE-MA	DMP
3-hydroxyisovaleric acid	1.02	1.27	1.21	0.35	0.51
L-citramalic acid	1.22	1.57	1.22	0.44	0.39
Formic acid	1.36	1.75	1.43	0.40	0.35
Fumaric acid	1.35	0.99	1.06	-	0.32
Trigonelline	0.97	1.47	1.89	-	0.48
N,N-dimethylglycine	1.14	1.57	0.96	0.40	-
Valine	1.01	1.44	1.08	0.30	-
Acetone	1.18	1.18	1.56	0.33	-
1-Methylhydantoin	1.05	1.26	1.12	0.34	-
Phenylalanine	0.88	0.91	0.95	-	0.35
Pyruvic acid	0.94	1.19	1.16	-	0.39
Propylene glycol	1.38	1.78	3.07	-	0.52
N-acetylaspartic acid	0.99	1.03	1.19	-	0.35
1-Methylguanidine	1.06	1.21	1.14	-	0.45
1-Methylnicotinamide	1.03	0.85	1.15	-	0.40
Succinic acid	1.36	1.44	1.47	-	0.53
Alanine	1.02	1.40	1.15	0.43	-
Allantoin	1.05	1.47	1.04	0.37	-
Citric acid	1.27	1.73	1.25	0.33	-
Acetic acid	2.35	3.37	2.65	0.45	-
Glycine	1.04	1.50	1.10	0.40	-
Leucine	0.85	1.54	0.96	0.32	-
Pantothenic acid	1.11	1.20	1.24	0.33	-
Arginine	1.18	1.43	1.02	0.38	-

FC: Fold change

^a^ Mean concentration of the compound in the ADHD children divided by the mean concentration of the compound in the control children

^b^ Mean concentration of the compound in children with high urinary HNE-MA levels divided by the mean concentration of the compound in children with low urinary HNE-MA levels

^c^ Mean concentration of the compound in children with high urinary DMP levels divided by the mean concentration of the compound in children with low urinary DMP levels

^d^ Spearman correlation

**Fig. 2:**
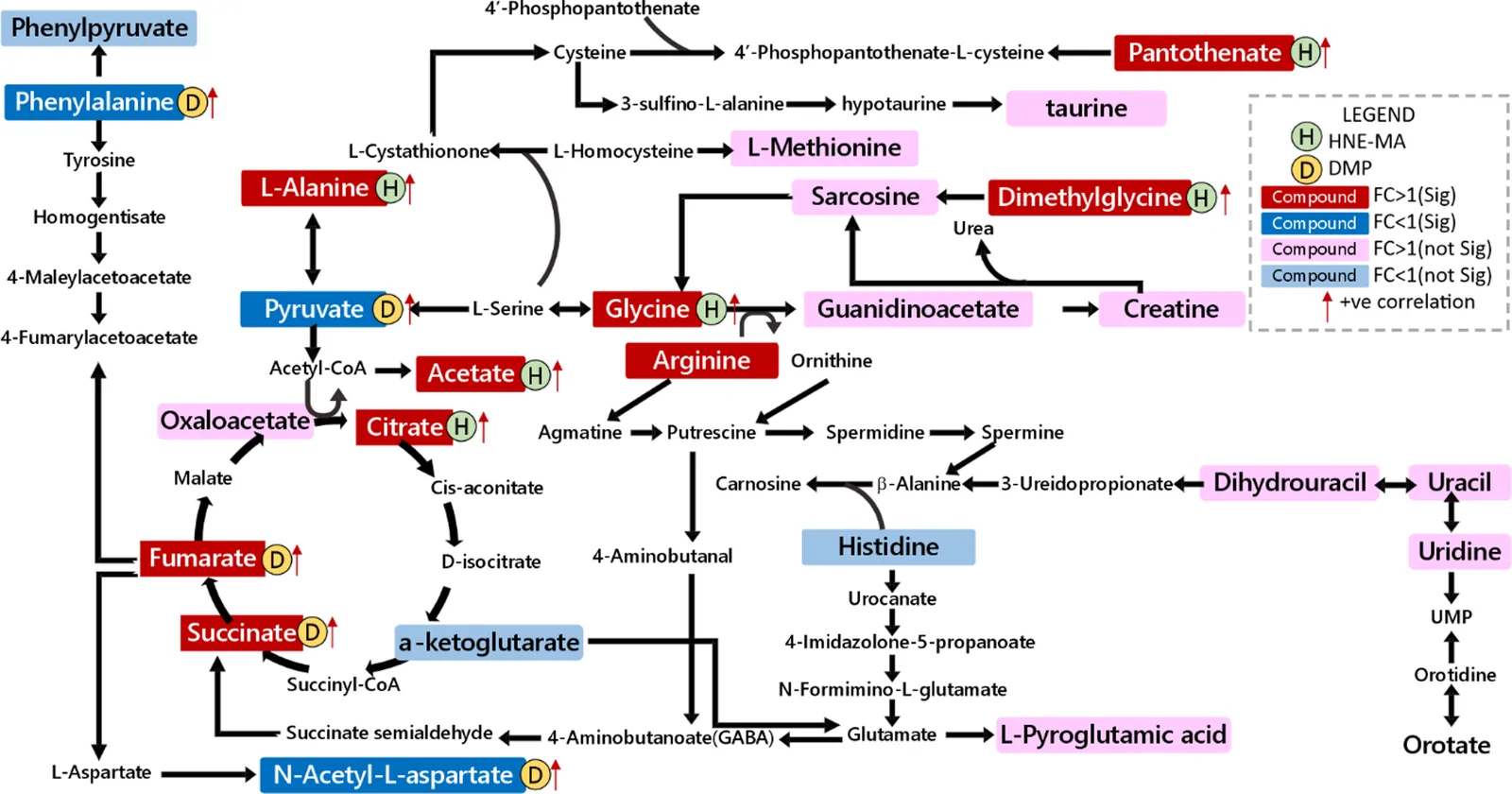
Metabolic pathways associated with ADHD in relation to OPs exposure and HNE-MA levels. ‘FC’ indicates fold change, which is the mean concentration of the compound in the ADHD children divided by the mean concentration of the compound in the control children. ‘Sig’ indicates the compound met the study significance criteria, while ‘Not sig’ indicates the compound was quantified but did not meet our study’s significant criteria. ‘+ve correlation’ indicates that the significant compound is positively correlated with HNE-MA or DMP

**Fig. 3:**
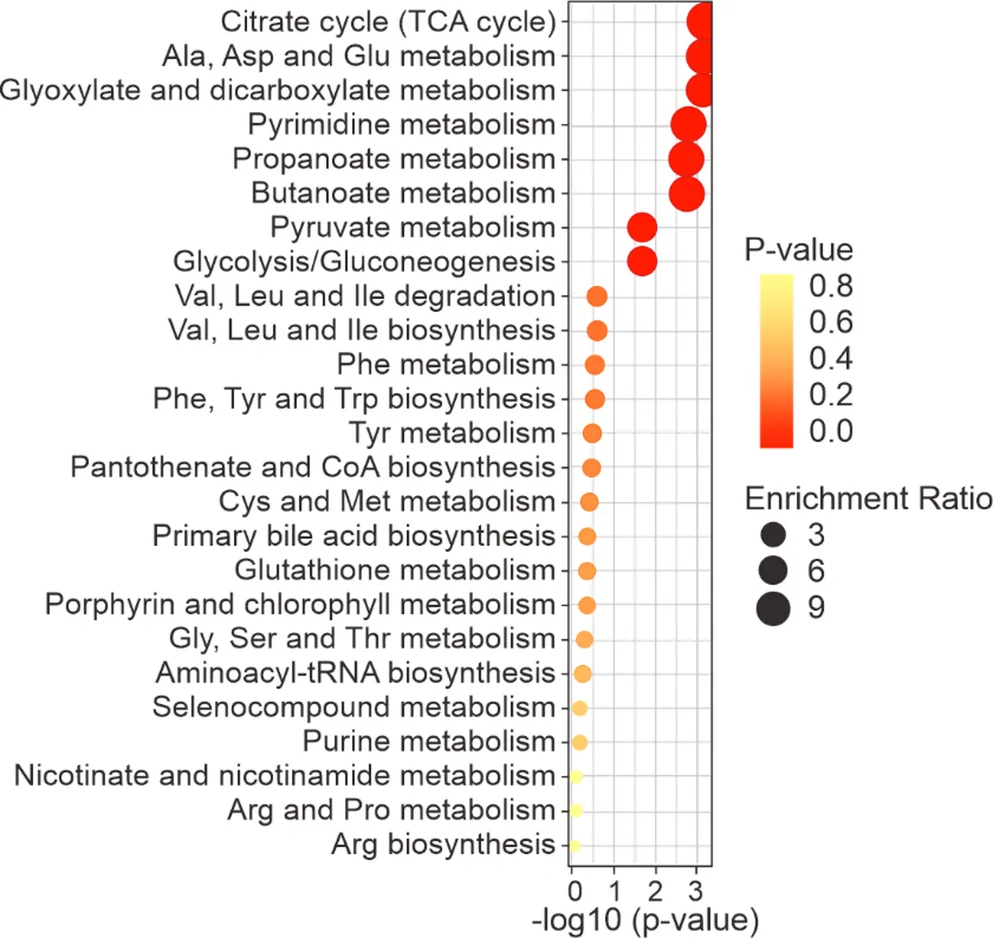
Metabolic pathway analysis plot. Color intensity (white to red) reflects increasing statistical significance, while circle diameter corresponds to pathway impact. The graph was obtained by plotting on the y-axis the − log of *p*-values from the pathway enrichment analysis and on the x-axis the pathway impact values derived from the pathway topology analysis

Among the 24 significant compounds identified by NMR-targeted analysis, 12 metabolites were mapped to human metabolic pathways in the KEGG database. These pathways are shown in Fig. 2, alongside their fold changes and correlations with HNE-MA and DMP. Urinary concentrations of compounds associated with the tricarboxylic acid (TCA) cycle were higher in ADHD children than in controls, and these compounds consistently showed positive correlations with urinary HNE-MA and DMP levels. Furthermore, urinary alanine, glycine, arginine, and dimethylglycine levels were elevated in ADHD children, whereas phenylalanine levels was lower in ADHD children compared with controls. Pathway analysis using MetaboAnalyst 5.0 highlighted the TCA cycle and the alanine, aspartate, and glutamate metabolic pathways as the most significantly perturbed pathways associated with ADHD (Fig. 3). In multivariable logistic regression analysis, urinary citric acid levels in ADHD children were significantly higher than those in control children, with an adjusted odds ratio of 4.286 (95% CI:1.235, 14.872) for ADHD after accounting for sex, and family history of nervous system diseases. Because creatinine concentration was already adjusted during sample preparation, it was not included again as a covariate in the regression models (Table S5).

**Fig. 4:**
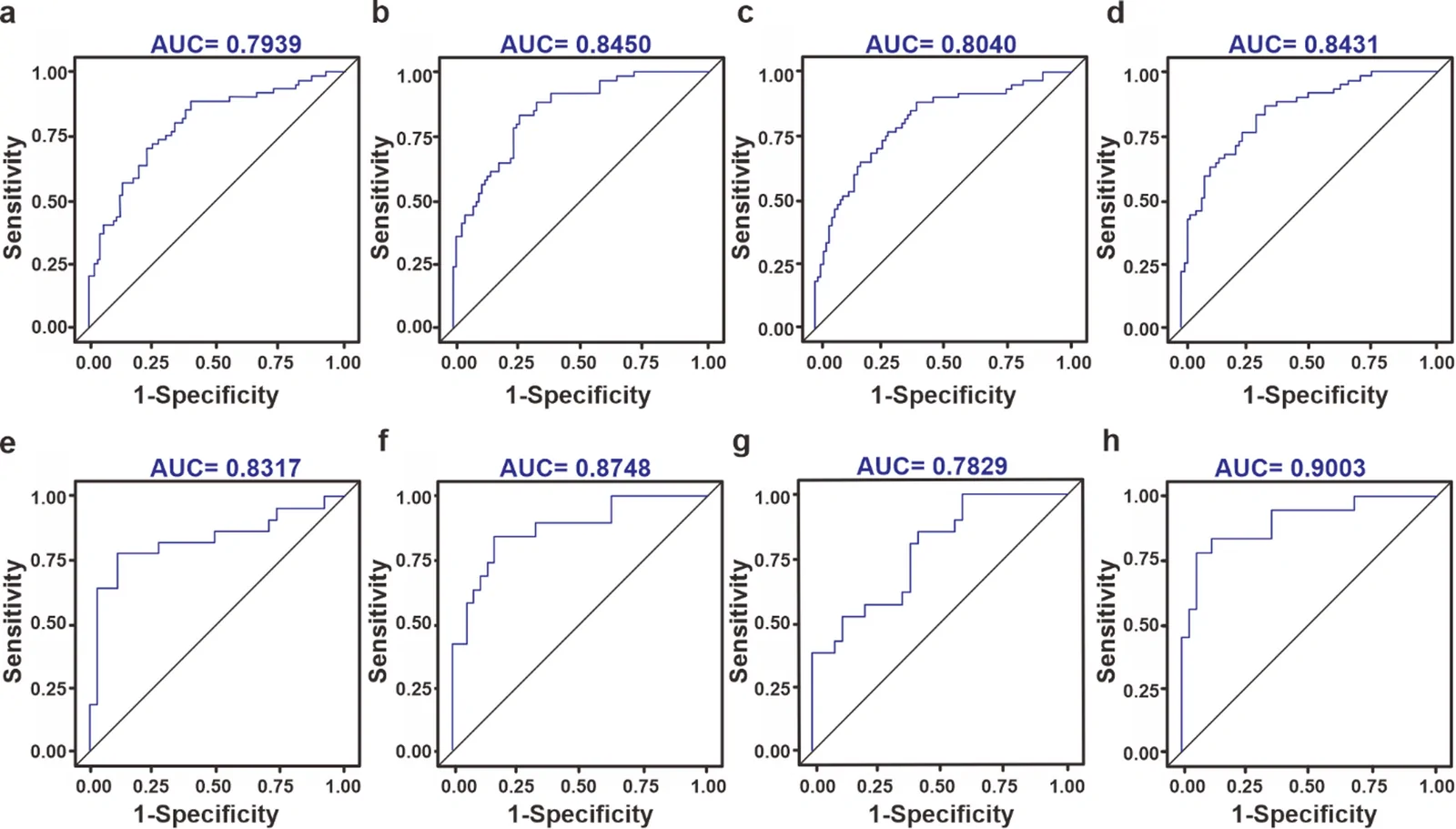
Receiver operating characteristic curve analysis of the predicted panel of all children in the discovery stage (**a**–**d**): **a** 10-compound panel, **b** 11-compound panel including HNE-MA, **c** 11-compound panel including DMP, **d** 12-compound panel including HNE-MA and DMP, and validation stage (**e**–**h**): **e** 10-compound panel, **f** 11-compound panel including HNE-MA, **g** 11-compound panel including DMP, **h** 12-compound panel including HNE-MA and DMP

Stepwise logistic regression was employed to select the 10 most significant compounds from a pool of 24 compounds as the ADHD-predictive model. In addition, three stepwise regressions were performed, incorporating HNE-MA and DMP into the model. The base model selected 10 metabolites; when HNE-MA or DMP was included, a similar pattern was observed (Table S6). The identified potential biomarkers include HNE-MA, leucine, acetic acid, arginine, trigonelline, valine, pyruvic acid, 3-hydroxyisovaleric acid, phenylalanine, N, N-dimethylglycine, and citric acid. The discriminatory performance of the stepwise logistic regression models, as measured by the AUC, is shown in Fig. 4. In the discovery stage, the 10-compound panel showed an AUC of 0.7939. When HNE-MA was added to the model, the discrimination slightly improved with an AUC of 0.8450 (Fig. 4a and b). The inclusion of DMP yielded an AUC of 0.8040, while the combined model, including both HNE-MA and DMP, resulted in an AUC of 0.8431 (Fig. 4c and d). In the validation cohort, the 10-compound panel achieved an AUC of 0.8317 (Fig. 4e), whereas the addition of HNE-MA and DMP in a separate 11-compound panel yielded AUCs of 0.8748 and 0.7829, respectively (Fig. 4f and g). The combined 12-compound panel with HNE-MA and DMP demonstrated the best predictive performance with an AUC of 0.9003 (Fig. 4h).

## Discussion

The urinary metabolite profiles of children in both the ADHD and control groups showed significant differences in the OPLS-DA score plots when stratified by high and low levels of HNE-MA and DMP. DMP reflects OPs exposure, whereas HNE-MA represents lipid peroxidation and oxidative stress. These two biomarkers represent distinct but related domains: external environmental exposure and internal biological response. The clustering results suggest that both OPs exposure and oxidative stress may contribute to metabolic dysregulation associated with ADHD.

DMP was selected as a biomarker of OPs exposure in this study based on our previous findings (Chang et al. 2018; Yu et al. 2016b). DMP had the highest detection frequency and concentration levels, and accounted for a large proportion of total DAP metabolites (Table S7). Among the six commonly measured DAP metabolites, DMP showed a significant positive association with ADHD and was the only metabolite associated with ADHD risk in the regression analysis. DMP concentrations were also strongly correlated with HNE-MA levels. HNE-MA was included as an endogenous biomarker of oxidative stress to supplement the assessment of OPs exposure and ADHD. Children with concurrently high DMP and HNE-MA levels showed a significantly higher risk of ADHD by exhibiting the highest adjusted odds ratio (AOR) than those with lower levels, suggesting a synergistic association between environmental exposure and oxidative damage (Table S7).

 The increased oxidative stress observed in this study may either result from inflammation associated with ADHD or contribute to its development. Previous studies have reported increased oxidative stress in children, adolescents, and adults with ADHD. Children with ADHD have been reported to have higher blood levels of total oxidants and altered antioxidant profiles than healthy children (Joseph et al., 2015; Sezen et al., 2016). Similar findings have also been observed in adolescents and adults with ADHD (Kul et al., 2015; Selek et al., 2012). One study suggested that oxidative stress parameters may have potential diagnostic value in ADHD (Selek et al., 2012). Our previous study found that among the four oxidative stress biomarkers, HNE-MA had the strongest association with ADHD risk and contributed most substantially to co-exposure effects (Chang et al., 2021; Waits et al., 2022). Higher oxidative stress in ADHD has also been linked to brain inflammation in patients (Corona, 2020). In addition, our previous study suggested that OPs exposure may interact with HNE-MA levels in relation to ADHD, indicating a complex relationship between OPs exposure, oxidative stress, and ADHD (Chang et al., 2021). These findings provide valuable insights into the metabolic alterations associated with ADHD.

The sociodemographic characteristics of the study population were comparable between the ADHD and control groups for age and BMI, although several other variables showed significant differences between the groups. A higher number of males in the ADHD group than females is consistent with the well-established male predominance of ADHD. Children with ADHD were more likely to have parents with lower educational levels and higher environmental tobacco exposure and maternal alcohol consumption; these factors have previously been associated with neurodevelopmental outcomes. The ADHD group also had a higher frequency of nervous system diseases in the family, suggesting a possible role of genetic susceptibility.

The predicted panel identified in this study was primarily composed of NMR-targeted compounds, allowing both qualitative and quantitative interpretation. The AUC of the prediction panel increased from 0.7939 to 0.8450 after the inclusion of HNE-MA. Although the outcome variables were set as ADHD and non-ADHD during the stepwise logistic regression, DMP was included in the analysis, allowing the selected metabolites to reflect the impact of OPs exposure on metabolic disturbance. DMP served as a predictor of OPs exposure, while HNE-MA represented an endogenous marker of oxidative stress. The 11-compound panel, including HNE-MA, may therefore reflect metabolic alterations closely related to ADHD, OPs exposure, and oxidative stress. Most of these compounds play significant roles in the human metabolic pathways. DMP and HNE-MA represent distinct but interconnected biological processes, such as OPs exposure and lipid peroxidation. The metabolic changes associated with DMP and HNE-MA were partially distinct, but their predictive capabilities for ADHD were similar, as indicated by AUC values. These findings suggest that metabolic pathway alterations in children with ADHD may be associated with both OPs exposure and HNE-MA, emphasizing the potential of these compounds as biomarkers.

The metabolic pathways examined indicate that children with ADHD exhibit a more active TCA cycle compared with controls, particularly among those with higher DMP exposure and higher oxidative stress. As a key energy production mechanism, the TCA cycle may contribute to ADHD-related hyperactivity, which could stem from increased energy demands, greater energy consumption, or excessive energy production. However, it remains unclear from urinary data alone whether this phenomenon is a cause or consequence of ADHD. The higher urinary citric acid levels observed in ADHD children further suggest possible alterations in the TCA cycle and mitochondrial function in ADHD (Akram, 2014).

In children with ADHD, urinary citric acid levels were significantly higher than those in controls after adjustment for covariates. The selection of covariates was based on their biological relevance. Elevated levels of alanine, essential for energy production, were also observed in ADHD children compared with controls, aligning with findings on pyruvate depletion in energy production in ADHD patients (Bornstein et al., 1990). Lower tyrosine levels have been reported in ADHD patients, suggesting potential disruptions in neurotransmitter synthesis, including dopamine and norepinephrine pathways (Bornstein et al., 1990). Although some studies reported no significant differences in aromatic amino acids between the ADHD and control groups (Bergwerff et al., 2016), urinary metabolites related to the tyrosine metabolic pathway showed substantial differences in patients with ADHD (Tian et al., 2022). The increased dimethylglycine (DMG) levels observed in ADHD children are also consistent with previous findings linking DMG levels with ADHD and nocturia (Yu et al., 2021). In addition, the association between betaine, a precursor of DMG, and oxidative stress further underscores these intricate metabolic alterations (Ohnishi et al., 2019). The metabolites identified within these pathways showed positive correlations with urinary HNE-MA and DMP levels.

Previous studies have shown that exposure to OPs could elevate oxidative stress levels and increase the risk of ADHD (Chang et al. 2018, 2021; Yu et al. 2016b). To mitigate excessive OPs exposure, thorough washing of fruits and vegetables and minimizing indoor use of insecticides have been recommended (Martin et al., 2022; Park et al., 2022). These measures align with broader approaches that consider both dietary habits and environmental exposure reduction.

To our knowledge, this study is the first to explore metabolic phenotyping in relation to exposure to OPs and ADHD in Taiwanese children. We used the SNAP-IV questionnaire for the diagnosis of ADHD, and pediatricians or psychiatrists further confirmed it based on the DSM-IV. There are several limitations in the present study. First, the use of single-spot urine samples may not adequately reflect long-term exposure to OP. Even though DMP is a predominant OP metabolite and a significant risk factor for ADHD, it could not fully represent the total OPs exposure. Second, the urinary metabolomic profiles observed in this study represent the exposure conditions between 2012 and 2015. Therefore, the present findings should be interpreted as metabolomic features associated with DMP and HNE-MA levels under exposure conditions during that period, and they may not reflect current OPs exposure levels. There may have been changes in pesticide regulation, agricultural practices, and human exposure patterns. Third, although the structured questionnaire allowed adjustment for covariates and strengthened the observed association, information provided by parents or guardians is subject to recall bias. Fourth, the limited sample size of females in this study led to greater intra-group differences, which may affect the interpretation of results, because OPs exposure may have sex-specific neurodevelopmental effects and metabolic outcomes. Therefore, the present findings should be interpreted as an overall and male-dominant metabolomic pattern rather than sex-specific metabolomic signatures. Fifth, the short half-life of OPs and temporal changes may influence urinary concentration. However, consistent lifestyle patterns among individuals may result in only slight variation in concentration. Finally, genetic, environmental, disease-related, nutritional, and medication-related factors may also influence metabolite profiles. In addition, other potential ADHD risk factors, including exposure to heavy metals such as lead, were not analyzed in this study.

In conclusion, the OPLS-DA-based grouping effectively distinguished high- and low-OPs exposure groups and groups with high and low HNE-MA levels within the ADHD and control groups. This study identified an 11-compound panel that may serve as a potential predictive biomarker panel for ADHD. Compounds identified within the TCA cycle may reflect differences in energy production and consumption and showed consistent positive correlations with urinary DMP and HNE-MA levels. These findings contribute to the growing understanding of the intricate interplay among OPs exposure, oxidative stress, ADHD, and metabolic alterations. However, because the metabolic alterations observed in this study reflect exposure conditions from 2012 to 2015, further studies using current exposure data are needed to determine whether these metabolomic features remain applicable to current exposure conditions. Larger studies are also recommended to enable stratified analyses and enhance statistical power. In addition, simultaneous analyses of blood and urine metabolites may provide a more comprehensive understanding of these associations.

## Supplementary Information

Below is the link to the electronic supplementary material.

Supplementary Material 1

## Data Availability

The datasets generated and/or analyzed in this study will be available upon request from the corresponding author.
