# Supplementary material for: Targeted and Untargeted Urinary Metabolomic Analyses of Organophosphate Pesticides Exposure and Attention-Deficit/Hyperactivity Disorder in Children
Source: Metabolomics. 2026 Jul 28;22(4):135. doi: 10.1007/s11306-026-02495-5 (PMC13415479; doi:10.1007/s11306-026-02495-5)
Supplement: Supplementary file 1 — Supplementary Material 1 [file 11306_2026_2495_MOESM1_ESM.pdf]

Supplementary information

Targeted and Untargeted Urinary Metabolomic Analyses of Organophosphate Pesticides Exposure and Attention-Deficit/Hyperactivity Disorder in Children

Metabolomics

Hsin-Yun Tseng[1#], Chi-Jen Lo[2, 3#], Boopathi Subramani[1], Jia-Woei Hou[4, 5], Ching-Jung Yu[1], Ting-Yu Fang[1], Betau Hwang[6], Mei-Ling Cheng[2, 3, 7, 8]*, Mei-Lien Chen[1]*

[1] Institute of Environmental and Occupational Health Sciences, School of Medicine, National Yang Ming Chiao Tung University, Taipei, Taiwan
[2] Metabolomics Core Laboratory, Healthy Aging Research Center, Chang Gung University, Taoyuan, Taiwan
[3] Clinical Metabolomics Core Laboratory, Chang Gung Memorial Hospital, Taoyuan, Taiwan
[4] School of Medicine, Fu Jen Catholic University, Taipei, Taiwan
[5] Department of Pediatrics, Cathay General Hospital, Taipei, Taiwan
[6] Department of Child and Adolescent Psychiatry, Taipei City Hospital, Taipei, Taiwan
[7] Department of Biomedical Sciences, College of Medicine, Chang Gung University, Taoyuan, Taiwan
[8] Graduate Institute of Biomedical Sciences, College of Medicine, Chang Gung University, Taoyuan, Taiwan

*Corresponding author: Prof. Mei-Lien Chen
No.155, Sec. 2, Linong Street, Taipei 112, Taiwan
Telephone: +886-2-28267239. Fax: +8862-2-8278254.
E-mail: mlchen@nycu.edu.tw

*Co-Corresponding author: Prof. Mei-Ling Cheng
No.259, Wenhua 1st Road, Guishan Dist., Taoyuan City 333, Taiwan
Telephone: +886-3-2118800 ext. 3811. Fax: +886-3-2118700.
E-mail: chengm@mail.cgu.edu.tw

#These authors contributed equally to this study.

Table S1. The grouping strategies of this metabolomics study.

M	Control[0], Case[1]	Male[0], Male[1]
HNE-MA Q1,<br>HNE-MA Q4	0HNE-MA Q1,<br>0HNE-MA Q4,<br>1HNE-MA Q1,<br>1HNE-MA Q4	M_0HNE-MA Q1,<br>M_0HNE-MA Q4,<br>M_1HNE-MA Q1,<br>M_1HNE-MA Q4
DMP Q1,<br>DMP Q4	0DMP Q1,<br>0DMP Q4,<br>1DMP Q1,<br>1DMP Q4	M_0DMP Q1,<br>M_0DMP Q4,<br>M_1DMP Q1,<br>M_1DMP Q4

’0’ indicates control, and ‘1’ indicates case. ‘Q1’ indicates low exposure, which means exposure level < 25th percentile. ‘Q4’ indicates high exposure, which means exposure level > 75th percentile.

Table S2. Demographic characteristics of all children

	ADHD N(%)	Control N(%)	p-value[a]
Total	88	134	
Age (year, mean ± SD)	8.79±2.81	8.88±1.95	0.500
BMI (kg/m^2, mean ± SD)	18.00±3.68	17.35±3.39	0.300
Sex			
Male	75 (85.2)	78 (58.2)	**<0.001**
Female	13 (14.8)	56 (41.8)	
Habit of sports in children			
No	40 (45.5)	40 (29.9)	**0.018**
Yes	48 (54.5)	94 (70.1)	
Maternal educational levels			
High school and below	36 (40.9)	39 (29.1)	0.069
College or higher	52 (59.1)	95 (70.9)	
Paternal educational levels			
High school and below	37 (42.0)	34 (25.4)	**0.009**
College or higher	51 (58.0)	100 (74.6)	
Exposure to environmental tobacco smoke			
No	51 (58.0)	97 (72.4)	**0.049**
Yes	37 (42.0)	36 (26.9)	
Missing		1 (0.7)	
Maternal smoking during pregnancy			
No	84 (95.5)	127 (94.8)	0.820
Yes	4 (4.5)	7 (5.2)	
Maternal drinking during pregnancy			
No	75 (85.2)	128 (95.5)	**0.007**
Yes	13 (14.8)	6 (4.5)	
Family history of nervous system disease			
No	58 (65.9)	110 (82.1)	**0.006**
Yes	30 (34.1)	24 (17.9)	
HNE-MA (μg/g creatinine)			
DR (%)	95.2	98.5	
GM (GSD)	39.67 (3.25)	19.01 (2.73)	
Median	35.31	16.73	**<0.001**
DMP (μg/g creatinine)			
DR (%)	100.0	98.5	
GM (GSD)	40.83 (2.00)	32.18 (2.58)	
Median	40.60	32.66	**0.046**

DR: Detection rate; GM: Geometric mean; GSD: Geometric standard deviation.

[a] Chi-square test

Table S3. Characteristics of children in the discovery stage compared to those in the validation stage.

	Discovery cohort N (%)	Validation cohort N (%)	p-value[a]
All	165	57	
ADHD	67 (40.6)	21 (36.8)	
Control	98 (59.4)	36 (63.2)	
Age (year, mean±SD)	8.96±2.39	8.52±2.09	0.30
BMI (kg/m^2, mean±SD)	17.42±3.35	18.10±3.93	0.20
Sex			
Male	113 (68.5)	40 (70.2)	0.81
Female	52 (31.5)	17 (29.8)	
Habit of sports in children			
No	58 (35.2)	22 (38.6)	0.64
Yes	107 (64.8)	35 (61.4)	
Maternal educational levels			
High school and below	54 (32.7)	21 (36.8)	0.57
College or higher	111 (67.3)	36 (63.2)	
Paternal educational levels			
High school and below	51 (30.9)	20 (35.1)	0.56
College or higher	114 (69.1)	37 (64.9)	
missing	51 (30.9)	20 (35.1)	
Exposure to environmental tobacco smoke			
No	113 (68.5)	35 (61.4)	0.49
Yes	51 (30.9)	22 (38.6)	
Missing	1 (0.6)		
Maternal smoking during pregnancy			
No	157 (95.2)	54 (94.7)	0.90
Yes	8 (4.8)	3 (5.3)	
Maternal drinking during pregnancy			
No	151 (91.5)	52 (91.2)	0.95
Yes	14 (8.5)	5 (8.8)	
Family history of nervous system disease			
No	151 (91.5)	52 (91.2)	0.95
Yes	14 (8.5)	5 (8.8)	

[a] Chi-square test

Table S4. Cumulative variation in the Y matrix (R^2Y) and the cross-validated predictive ability (Q^2) of the OPLS-DA models.

Model	Positive mode		Negative mode	
	R^2Y	Q^2	R^2Y	Q^2
Case Control x HNE-MA exposure [a] (all children)	0.846	0.899	0.852	0.928
Case Control x HNE-MA exposure [a] (male)	0.902	0.854	0.917	0.875
Case Control x DMP exposure [b] (all children)	0.849	0.908	0.847	0.922
Case Control x DMP exposure [b] (male)	0.922	0.902	0.911	0.856

[a] OPLS-DA score plots between case, control, low, and high urinary levels of HNE-MA in the discovery cohort.

[b] OPLS-DA score plots between case, control, low, and high urinary levels of DMP in the discovery cohort.

Table ~~S5~~S5. Adjusted odds ratio and the 95% confidence intervals of ADHD of the significant compounds.

	Odds Ratio[a]	Lower 95%CI	Upper 95%CI
Propylene glycol	1.882	0.951	3.727
1-Methylguanidine	1.169	0.195	7.020
Alanine	2.361	0.496	11.248
Arginine	0.470	0.198	1.116
Glycine	1.719	0.480	6.152
Leucine	0.453	0.168	1.223
N, N-Dimethylglycine	1.076	0.238	4.871
N-Acetylaspartic acid	0.660	0.022	19.751
Phenylalanine	0.691	0.354	1.348
Valine	1.112	0.220	5.610
Acetic acid	14.883	**2.569**	**86.227**
Citric acid	4.286	**1.235**	**14.872**
Fumaric acid	4.071	0.861	19.241
Succinic acid	8.261	**2.254**	**30.273**
Trigonelline	0.406	0.141	1.173
Pantothenic acid	1.932	0.197	18.979
3-Hydroxyisovaleric acid	1.212	0.130	11.309
L-Citramalic acid	3.479	0.778	15.570
Acetone	1.242	0.322	4.799
Pyruvic acid	0.620	0.075	5.143
1-Methylhydantoin	1.300	0.288	5.872
1-Methylnicotinamide	1.591	0.362	7.001
Allantoin	0.736	0.200	2.701

[a] Logistic regression

Table ~~S6~~S6. Logistic stepwise regression of all children in the discovery stage.

Step	Effect	Number in[a]	Score Chi-Square	P-value
1	Acetic acid	1	12.953	0.000
2	Arginine	2	7.730	0.005
3	N-Acetylaspartic acid	4	4.655	0.031
4	Trigonelline	5	3.159	0.076
5	Citric acid	6	3.824	0.051
6	Pyruvic acid	7	2.608	0.106
7	3-Hydroxyisovaleric acid	8	4.723	0.030
8	Formic acid	9	3.145	0.076
9	N, N-Dimethylglycine	10	2.695	0.101
10	Pantothenic acid	11	2.381	0.123
**Inclusion of HNE-MA**				
1	Leucine	2	9.326	0.002
2	Acetic acid	3	8.915	0.003
3	Arginine	4	7.427	0.006
4	Trigonelline	5	3.163	0.075
5	Valine	7	2.573	0.109
6	Pyruvic acid	8	4.369	0.037
7	3-Hydroxyisovaleric acid	9	4.684	0.031
8	Phenylalanine	10	2.344	0.126
9	N, N-Dimethylglycine	11	2.686	0.101
10	Citric acid	12	3.222	0.073
**Inclusion of DMP**				
1	Acetic acid	2	10.470	0.001
2	Arginine	3	7.661	0.006
3	Trigonelline	5	6.548	0.011
4	Pyruvic acid	6	4.834	0.028
5	3-Hydroxyisovaleric acid	7	5.648	0.018
6	Citric acid	8	3.132	0.077
7	Formic acid	9	2.818	0.093
8	L-Citramalic acid	10	2.070	0.150

9	Valine	11	1.762	0.184
10	Acetone	12	2.108	0.147
Inclusion of HNE-MA and DMP				
1	Leucine	3	8.815	0.003
2	Acetic acid	4	8.059	0.005
3	Arginine	5	7.486	0.006
4	Trigonelline	6	3.929	0.048
5	Pyruvic acid	8	3.295	0.070
6	3-Hydroxyisovaleric acid	9	4.890	0.027
7	Valine	10	3.495	0.062
8	L-Citramalic acid	11	2.401	0.121
9	Citric acid	12	3.693	0.055
10	N, N-Dimethylglycine	13	2.025	0.155

[a]The order of compounds was entered (selected).

Table ~~S7~~S7. Distribution of urinary DMP and DAP metabolites and oxidative stress biomarkers and the risk of ADHD among children.

	DMP[a, c]		DAP[a, c]		HNE-MA[b, d]	
	ADHD	Control	ADHD	Control	ADHD	Control
**Distribution**						
Detection rate	100	97.9	-	-	100	100
GM (SSD)	238.95 (2.11)	164.83 (2.35)	553.49 (2.08)	444.68 (2.10)	30.75 (3.11)	18.41 (2.91)
Range	22.98-2306.18	3.25-875.62	126.88-4817.58	49.42-2787.8	2.62-432.56	2.21-478.89
25%	138.81	107.81	316.3	273.13	12.84	9.02
50%	250.78	173.91	540.15	446.23	27.43	15.82
75%	366.33	302.86	848.39	708.43	72.43	27.99
p-value	**0.01**		0.12		**0.01**	
**ADHD Risk**						
ORs (95% CI)	1.84 (1.23-2.77)*		1.52 (1.00-2.26)*		1.53 (1.16-2.02)*	
AORs (95% CI)	2.37 (1.30-4.32)*		-		1.73 (1.19-2.52)*	
ORs (95% CI)	-		-		1.61 (1.15-2.28)*	
AORs (95% CI)	-		1.46 (0.87-2.45)		1.58 (1.13-2.20)*	

GM: geometric mean; GSD: geometric standard deviation; Concentrations less than LOD were replaced with values equal to LOD/2.

[a]: nmol/g cre.; [b]: µg/g cre.

[c]: Concentration of the natural log transformation (nmol/g cre.); [d]: Concentration of the natural log transformation (µg/g cre.)

*: p value<0.05; ORs: odds ratios; AORs: adjusted odds ratios; CI: confidence interval; adjusted covariates: age, sex, BMI, maternal education (reference: high school and below), alcohol consumption during pregnancy (reference: did not consume alcohol), environmental tobacco smoke exposure (reference: no exposure), and family history of nervous system diseases (reference: no family history).
